# A Survey of Routing Protocols in WBAN for Healthcare Applications

**DOI:** 10.3390/s19071638

**Published:** 2019-04-05

**Authors:** Yating Qu, Guoqiang Zheng, Huahong Ma, Xintong Wang, Baofeng Ji, Honghai Wu

**Affiliations:** 1 School of Electronic Information Engineering, Henan University of Science and Technology, Luoyang 471023, China; 170317050197@stu.haust.edu.cn (Y.Q.); mhh@haust.edu.cn (H.M.); 180318050225@stu.haust.edu.cn (X.W.); baofengji@haust.edu.cn (B.J.); honghai2018@haust.edu.cn (H.W.); 2 Henan Key Laboratory for Machinery Design and Transmission System, Henan University of Science and Technology, Luoyang 471003, China

**Keywords:** wireless body sensor networks, routing classification, energy efficiency, temperature, posture, cluster

## Abstract

The emergence of wireless body area network (WBAN) technology has brought hope and dawn to solve the problems of population aging, various chronic diseases, and medical facility shortage. The increasing demand for real-time applications in such networks, stimulates many research activities. Designing such a scheme of critical events while preserving the energy efficiency is a challenging task, due to the dynamic of the network topology, severe constraints on the power supply, and the limited computation power. The design of routing protocols becomes an essential part of WBANs and plays an important role in the communication stacks and has a significant impact on the network performance. In this paper, we briefly introduce WBAN and focus on the analysis of the routing protocol, classify, and compare the advantages and disadvantages of various routing protocols. Lastly, we put forward some problems and suggestions, which provides ideas for the follow-up routing design.

## 1. Introduction

Wireless body area networks (WBAN) is a branch of wireless sensor networks (WSN), and has attracted much attention because of its huge potential value. The emergence of WBAN can alleviate or even solve some social problems [1], such as various rampant chronic diseases, increasing aging population, tense medical personnel and facilities, etc. Therefore, more and more people expect the WBAN technology to be applied, as soon as possible. Although the rapid development of sensor technology and communication technology has led to the progress of WBAN, the technology is still in its infancy, and there are still many problems and challenges in its research and application [2].

This paper investigates and analyzes the existing range of studies, and introduces the basic architecture, application fields, and characteristics of the WBAN. It is well-known that one of the difficulties in the design of WBAN is the energy constraints [3]. To solve this problem, we can consider the two directions of open source and throttling. The open source method is a variety of wireless energy collection technologies, and the throttling method is a variety of energy efficiency design. Reasonable routing design is also one of the energy efficiency methods, so the object of this paper is to study a variety of routing. Routing is responsible for establishing the path in the network, connecting the scattered nodes into a whole, and realizing the timely and reliable transmission of data. The design principles of routing are stability and energy efficiency, to maximize the energy efficiency of a single node, as far as possible. Thus, it is very important to study the routing of WBAN. Through the analysis and summary of the vast literature, the routing protocols of WBAN can be classified as—posture-based routing, temperature-based routing, cross-layer routing, cluster-based routing, and Qos-based routing. On this basis, the advantages and disadvantages of each kind of routing protocol are analyzed, which provides direction and ideas for further research. In addition, the paper discusses the current and future issues of routing design in the prospect section, providing readers with reference and new ideas, which is the biggest difference from other reviews.

## 2. Introduction of Wireless Body Area Network

### 2.1. WBAN Architecture

**Figure 1:**
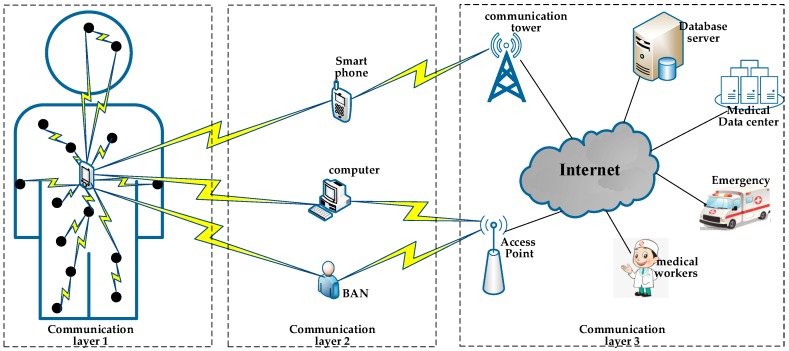
Network architecture of a Wireless Body Area Network (WBAN).

With the rapid development of the wireless communication technology and sensor technology, Wireless Body Area Network (WBAN) has been developed as a new technology. WBAN is a three-tier architecture body network [4], as shown in Figure 1. The tier-1 is composed of sensors attached to the body surface or implanted into the body. Its function is to collect and transmit various physiological information about the human body. The tier-2 is composed of smart phones, personal computers, or other intelligent electronic devices. The information sent by sensors is forwarded to the terminal data center by a wireless mode. In tier-3 of WBAN, the terminal data center is mainly composed of remote servers providing various applications. Its function is to collate and analyze the received data, to provide a dynamic response. Especially when the sensor node collects abnormal data, it will carry out emergency transmission and alarm, which can speed up the emergency processing and rescue.

### 2.2. Application Field of a Wireless Body Area Network

**Figure 2:**
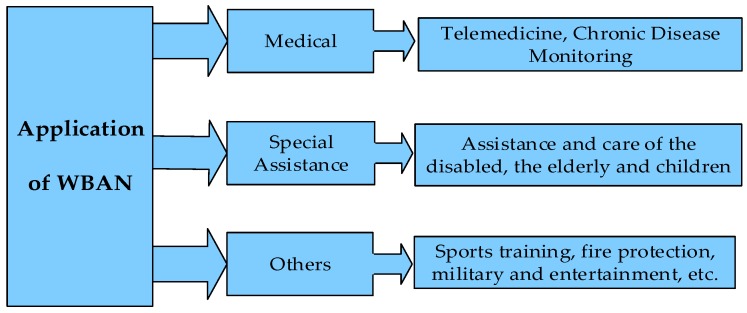
Applications of WBAN.

WBAN takes the human body as the application carrier, turns it into a part of the communication network, truly realizes the ubiquitous network and omnipresent service, and plays an important role in the following areas [5], as shown in Figure 2:

- Medical care services and chronic disease surveillance: These are the most important applications. There has been no good solution for the prevention and treatment of chronic diseases, worldwide. The emergence of WBAN brings hope for the treatment and prevention of various chronic diseases. Through the real-time comprehensive analysis and processing of the collected physiological information, we can effectively prevent and treat the disease, before and during onset. We can also save the physiological information of the disease for subsequent diagnosis, treatment, and medical research. The most important thing is that patients can get rid of the trouble of wired monitoring and experience the convenient service of remote medical monitoring [6].
- Assistance for special groups, such as the disabled and the elderly: Sensor devices are worn on the limbs of blind people, which can perceive the surrounding environment and road information, in real-time, to provide navigation and position services for blind people. Developing a care system for the elderly which can sense the actions of the elderly and make corresponding reminders, by locating the elderly, and record the activities to provide security and safety of the elderly.
- Other applications: Building human body network on athletes, real-time monitoring of athletes’ physical condition, during training, and changing training intensity at any time; providing fire fighters with special environment monitoring (such as fire scene, toxic gas environment, etc.), warning firefighters regarding their own safety when their endurance threshold has exceeded; and, in the military, transferring important military intelligence to control centers or remote command troops, through wireless means. With the development of sensor technology and wireless network transmission technology, WBAN will be more and more widely used in medicine, including health monitoring, sports, entertainment, military, and aerospace fields, in the future. It also has considerable social value and economic benefits.

### 2.3. Characteristics of the Wireless Body Area Network

WBAN is a body network where a lot of sensor nodes are placed in, on or around the human body, to sense data, which is then transmitted through wireless means, to provide continuous and uninterrupted services [7]. WBAN is a special application branch of WSN—they have similarities but many differences. A detailed comparison of the two is shown in Table 1 [8].

### 2.4. Challenges in WBAN Routing Protocol Design

In view of the particularity of WBAN, the IEEE 802.15 working group set up a TG6 working group for WBAN, in 2007, to study the standardization of the WBAN technology. In 2012, the first WBAN standard proposed in the world, IEEE802.15.6, was officially released. The formulation of this standard also promoted the rapid development of WBAN.

WBAN is a technology applied to human body, so its network environment is very complex. Since the physiological data collected have a great impact on human life and health, it is very important to design routing for the body area network. Based on the above analysis, the following problems and challenges must be considered in routing design [9,10]:

1. Dynamic changes of the topological structure: Wireless transmission involves body surface transmission, body transmission, and free space transmission. Channel conditions are complex. Shadow effect caused by human motion should also be considered. The distance and relative position between nodes will also change with the movement of limbs. Considering the time-variation of the topology and the possible problems [11], reliable routing mechanism should be designed to adapt to the dynamic topology.
2. Energy efficiency: WBAN is a technology applied in human body, some sensors are implanted in the body and must be replaced by surgery. It is not enough to supply power only through micro-batteries. Now, although RF (Radio Frequency), EM (Electro Magnetic) or energy harvesting can be used for the power supply, energy efficiency design should also be carried out at the source. Therefore, energy efficiency of single node and energy balance of the whole network must be considered in the routing design, so as to prolong the lifetime of the network, as far as possible.
3. Node temperature: Nodes generate heat when they work, which can cause damage to the key tissues and organs of the human body [12,13]. Therefore, the temperature of nodes must be taken into account in the routing design, to avoid this kind of situation.
4. Various Qos requirements: Nodes in the WBAN generate different types of data, which must be processed differently, to ensure the Qos requirements of different types of data, such as emergency data, delay sensitive data, reliability sensitive data, and general data.

## 3. Classification of Routing Protocols for WBAN

**Figure 3:**
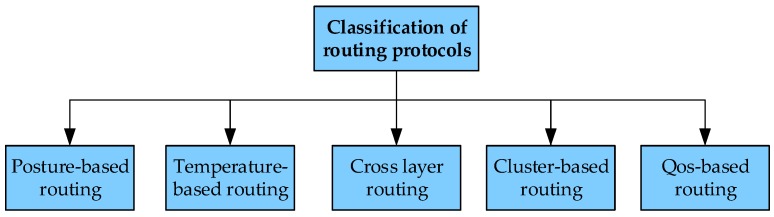
Routing classification for wireless body area networks.

At present, the research on routing protocols for WBAN is mainly divided into the following methods [14,15], and this paper refers to the classification method presented by Bangash et al. [14], (as shown in Figure 3) posture-based routing, temperature-based routing, cross-layer routing, QoS-based routing, and cluster-based routing.

### 3.1. Posture-Based Routing

Posture-based routing is used to analyze the network topology of the human body in various dynamic postures to establish a fast and stable path. The analysis of various postures is of great significance. Many experiments have proved that all kinds of human body movements have some regularity [16]. This can greatly improve the development of the dynamic WBAN that is used. In the dynamic environment, the movement of limbs make the network topology good or bad, and also produce a shadow effect. The existing solutions can only be further processed, after the situation occurs, which was very passive and had a large delay. If the next action is predicted in advance in the current time-slot, the judgment and processing can be made in advance, which is very helpful to reduce the delay and improve the data transmission rate, successfully [17].

#### 3.1.1. A Novel Mobility Handling Routing Protocol (MHRP)

Karmakar et al. [18] proposed a novel mobile processing routing protocol MHRP. The protocol is designed for heart rate acquisition. Seven nodes are located on the left and right sides of the human body. It is a set of two symmetrical nodes, each set has one sink node, two relay nodes and one common acquisition node. A new type of fault-tolerant system is composed of two identical and independent sets of nodes. The purpose of the design is to change another group, when the current working set energy is scarce, or the topology is interrupted, so as to reduce errors and improve the reliability of the data transmission.

**Figure 4:**
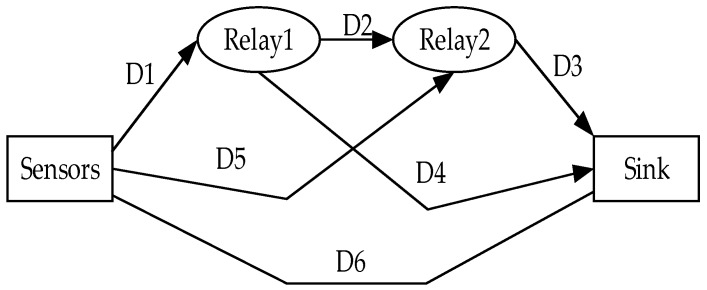
Routing Node Arrangement Diagram of MHRP Protocol.

The MHRP protocol makes network topology analysis for four kinds of elbow actions, and finds the best path for the four kinds of actions. As shown in Figure 4, although the relative position of the nodes will change with the elbow actions, there are only four paths at all times:

(1) $$\begin{array}{l} (\Delta 1)=D1+D2+D3\\ (\Delta 2)=D1+D4\\ (\Delta 3)=D5+D3\\ (\Delta 4)=D6 \end{array}$$

In path planning, the smallest one in Δ1,Δ2,Δ3,Δ4 is the current path, in which the temperature and residual energy of nodes are taken into account. The protocol divides data into general and emergency. When it is an emergency data, it uses Δ4 path to transmit directly, and when it is general data, it chooses relay transmission. Finally, the simulation results show that the protocol has a great improvement in the end-to-end delay, error rate, and energy consumption. However, the possible disadvantage is that the comfort of human body cannot be fully considered. Although the form of substitution set can achieve a stable performance, it increases the burden on the human body.

#### 3.1.2. Energy-Efficient and Distributed Network Management Cost Minimization (NCMD)

**Figure 5:**
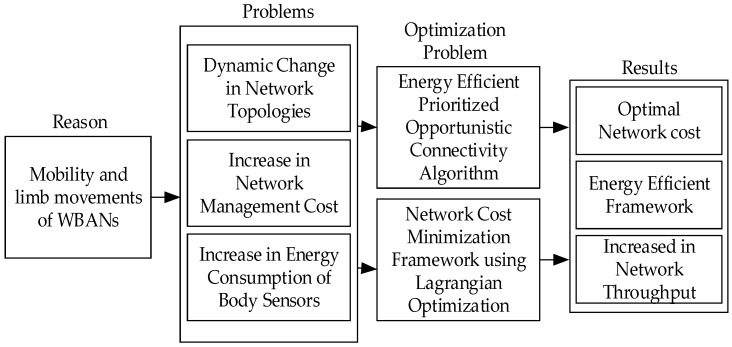
Overall framework of the NCMD protocol.

An efficient and distributed opportunity routing NCMD with minimal network management costs was proposed by Samanta et al. [19]. Experiments show that when WBAN is in a dynamic scene, the network topology is always in an intermittent and continuous state, which causes large transmission delay and higher network maintenance costs. Additionally, with the increase of mobile speed, various Qos indicators become worse and worse. To solve the above problems, this paper proposes an opportunistic transmission link establishment algorithm, to minimize the cost of network management and optimize Qos. The overall framework of the protocol is shown in Figure 5.

Through a lot of theoretical deduction and analyses, this paper establishes an optimal opportunity dynamic connection cost function, by using the price-based method, as follows:

(2) $$c_{OC_{tot}}^{t}=\sum_{i=1}^{N}\sum_{j=1}^{M}\Gamma_{ij}\left[1+\frac{\beta_{ij}^{t}}{\beta_{\max}}(c_{opp}^{t}-c_{opp_{th}}^{t})\right]$$

where $\Gamma_{ij}$ represents the scale factor, $\frac{\beta_{ij}^{t}}{\beta_{\max}}$ represents the instantaneous link quality factor of the nodes and the sink node, and $c_{opp_{th}}^{t}$ is the threshold of opportunistic dynamic connection. Then the total network management cost-function is deduced to minimize the evaluation, as follows:

(3) $$c_{tot}^{t}=c_{OC_{tot}}^{t}+c_{DC_{tot}}^{t}+c_{inff_{tot}}^{t}+c_{qos_{tot}}^{t}+c_{E_{tot}}^{t}+c_{DT_{tot}}^{t}$$

(4) $$Minimize\;u=\sum_{t=1}^{T}c_{tot}^{t}$$

(5) $$Subject\;to\;\sum_{t=1}^{T}c_{OC_{tot}}^{t}\geq c_{OC}^{th},c_{qos_{tot}}^{t}\geq c_{qos}^{th},c_{inff_{tot}}^{t}\geq c_{inff}^{th}$$

where $c_{OC_{tot}}^{t}$ is the cost of connection, $c_{DC_{tot}}^{t}$ is the cost of data transmission, $c_{inff_{tot}}^{t}$ is the cost of interference management, $c_{qos_{tot}}^{t}$ is the cost of Qos management, $c_{E_{tot}}^{t}$ is the cost of energy management, and $c_{DT_{tot}}^{t}$ is the cost of dynamic topology management. $c_{DT_{tot}}^{t}$, $c_{DT_{tot}}^{t}$, and $c_{inff}^{th}$ are the corresponding thresholds respectively. Lastly, the Lagrange operator is used to optimize the cost function of the network management. Based on the above theoretical analysis and simulation, the data show that the protocol is more adaptable in the dynamic WBAN environment, can quickly establish the connection path in the case of a link failure, reduce the cost of the network management, and improve the stability of the network.

#### 3.1.3. Comparison and Analysis

As shown in Table 2, the above two protocols are all modeled in the dynamic WBAN environment. The difference is that the MHRP [19] protocol monitors the heart condition, under the condition of human body movement, which uses two identical and independent sets of nodes to form a fault-tolerant system. The method is novel, but its function is too single, which achieves a reliable transmission by redundant nodes, the relay node only has the function of receiving and forwarding, to balance the energy consumption of the acquisition node. It cannot fully consider the human comfort, which can bring inconvenience to people’s normal life. NCMD [19] takes full account of the uncertainty of the topological connections in dynamic environments, uses the opportunity to establish connections in a targeted manner, moreover, it minimizes the cost of network management and improves the energy efficiency. By contrast, NCMD is more adaptable to the environment of dynamic WBAN.

### 3.2. Temperature-Based Routing

**Figure 6:**
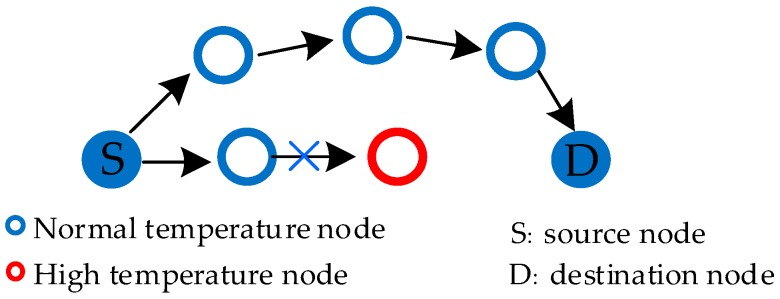
Temperature-sensing routing.

Temperature-based routing considers the temperature of nodes as the main parameter in the process of path selection. The main purpose of this routing method is to avoid the temperature rise of nodes, or quickly reduce the temperature of high-temperature nodes, by avoiding high-temperature nodes and establishing appropriate paths [20]. The routing schematic is shown in Figure 6. Temperature-based routing is the first method to be studied, because the security problem is the first one to be solved in the application of WBAN. Temperature-based routing has been widely studied during the early developmental period of WBAN, but in recent years, a large number of studies have focused on energy, so the temperature-based routing process has been slightly reduced.

#### 3.2.1. Thermal-Aware Routing Algorithm (TARA)

The TARA protocol proposed in Tang et al. [21] is the earliest achievement proposed in this field and is also a classic work. This method takes temperature as the only parameter of path selection and chooses the neighbor node with the lowest temperature as the next hop. Data sent to the hotpot are cached, which then waits for the destination node to cool down and is then retransmitted; if the waiting time exceeds a certain value, then the data are discarded. When data encounters a hotpot in transmission, the retreat strategy is adopted to return the original data path to find a new path. The back-off strategy used in the protocol, not only achieved good performance at that time, but also provided ideas for future research.

#### 3.2.2. A New Energy-Efficient Routing Protocol (ER-ATTEMPT)

**Figure 7:**
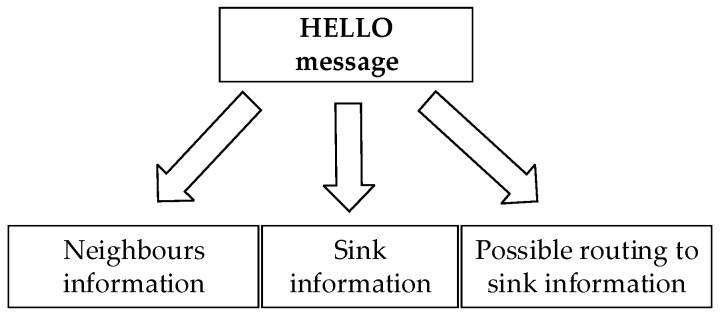
HELLO message format of ER-ATTEMPT protocol.

Ahmad et al. [22] proposed an ER-ATTEMPT protocol, which not only avoids the nodes with high temperature, but also chooses the path with the minimum hops as the best route. This protocol is an optimization of the ATTEMPT protocol, as the protocol of ATTEMPT does not take into account the unavailability of the current path, and ER-ATTEMPT protocol is optimized on this basis. In the initial stage, the node knows all the available paths by exchanging HELLO messages. If the current path is not available, then it chooses the sub-optimal path as the transmission path to ensure a reliable transmission. The message format is shown in Figure 7.

ER-ATTEMPT protocol also pays attention to the deployment of nodes, nodes are placed orderly, according to the level of energy. Initially, the energy is unbalanced, and relay nodes are equipped with more energy, which makes it easier to forward data from other nodes. In the routing stage, when the node generates normal data, relay transmission is used to find the best path, with the least hops, in the optional routing table. When the node produces abnormal data, the emergency transmission mode, which communicates directly with the sink node, is adopted. This routing method can not only ensure the timely transmission of emergency data, but also realize the reliable transmission of general data. However, there may be some problems—direct communication with sink will increase the probability of packet loss, due to long distance, and will also consume a lot of energy. It is not the best choice to ensure low latency at the cost of energy and reliability.

#### 3.2.3. Trust and Thermal Aware Routing Protocol (TTRP)

A trust and heat-aware routing protocol TTRP was proposed by Bhangwar et al. [23]. This paper considered two parameters—trust and temperature. Different from other routing methods, this routing method adds additional relay nodes, which only have the function of receiving and forwarding, do not participate in information collection, and are equipped with a higher energy to complete the function of forwarding information from other nodes.

**Figure 8:**
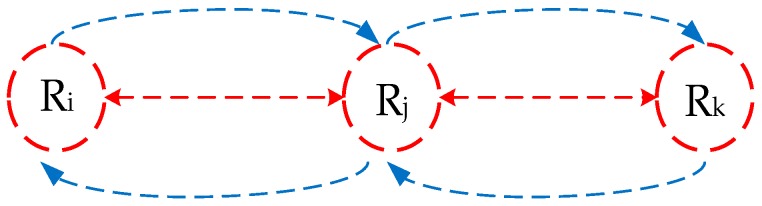
TTRP protocol evaluation trustworthiness diagram of relay node R_j_.

TTRP protocol includes three stages—trust estimation stage, routing discovery stage, and routing maintenance stage. The stage of trust estimation is mainly responsible for estimating the trustworthiness of the relay nodes (as shown in Figure 8), specifically through estimation of the buffer of R_i_ and R_k_ nodes, to determine whether the R_j_ nodes are untrusted nodes. Routing phase is to find the best path without the hotpot and the trusted nodes. TTRP constructs a composite function to help the routing phase find a reliable path, quickly and accurately. The optimal path is the path with the smallest value of the composite function. The composite function is shown in (6):

(6) $$CF=\omega_{1}\times Trust+\omega_{2}\times Temp$$

where $\omega_{1}$ and $\omega_{2}$ are the proportion weights of trust and temperature, respectively, and $\omega_{1}+\omega_{2}=1$. The two weight factors can be changed, according to the actual situation, and the respective proportion of trust and temperature can be adjusted accordingly. TTRP uses two parameters of temperature and trust to find the best routing, which not only avoids the node with high temperature to become the current routing, but also distributes the network load and reduces the probability of nodes becoming hotpot, thus, ensuring the reliable transmission of node data. However, there may be a problem that ignores energy consumption and delay.

#### 3.2.4. A Mobility-Based Temperature-Aware Routing Protocol (MTR)

Kim et al. [24] proposed a temperature-aware routing protocol MTR, based on mobility. The existing temperature-based routing does not take into account the changes of human posture and mobility. Topological separation will lead to a large number of data loss. This protocol is optimized and improved to solve the above problems. It not only solves the temperature problem, but also uses the storage and carrying mechanism of the DTN network to solve it. The problem of data packet loss caused by topological separation is, thus, solved. The protocol divides the nodes of the body area network into two categories—static nodes and dynamic nodes. Static node refers to the nodes located in the center of the human body that are not affected by human movement, while dynamic node refers to nodes located in the mobile position of a human arm or leg.

In the routing stage, the routing process of the static node is as follows—if there is a connected path between the node and the sink node, then it will communicate directly with the sink; otherwise, the temperature of the neighbor node and the connection probability of the dynamic node will be calculated, and the dynamic neighbor node with low temperature and high connection probability, will be selected as the next hop, then the data packet will be sent directly to the sink node, by the movement of the dynamic node. The routing process of dynamic nodes is as follows—when the current node is a dynamic node, all data are cached, if the connectivity between the current node and the sink node is detected, data are sent directly to the sink node, otherwise the cache time of the data packet are updated; when the cache time exceeds a certain value, no waiting is needed to find the static neighbor node with the lowest temperature, and if it exists, then the data are transferred to the section. Then the routing process is repeated for the static nodes.

#### 3.2.5. Comparison and Analysis

The above routing protocols are focused on solving the node temperature. A simple comparison of the above protocols is made. The comparison of the objectives, characteristics, complexity, delay, and energy efficiency of the protocols is shown in Table 3.

ER-ATTEMPT [22] and TTRP [23] protocols are built into the static WBAN environment, while MTR [24] considers a dynamic WBAN environment. MTR protocol is more practical to divide network nodes into static and dynamic nodes, and use the mobility of dynamic nodes to complete data forwarding, which is innovative and is worth learning.

When choosing the next hop, ER-ATTEMPT and TTRP have similarities, and both construct cost functions, but TTRP’s cost functions are more flexible than using weighting factor to weigh two parameters that can achieve different performance, its method is worth learning.

### 3.3. Cross-Layer Routing

Cross-layer routing protocol mainly integrates multiple protocol layers, and integrates the advantages of each protocol stack to achieve a better network performance. Experiments showed that the cross-layer method is more adaptable to dynamic WBANs, and the collaboration between different layers can better serve different priority data, provide customized services for each type of data, and achieve a comprehensive network performance with a low latency, high reliability, and energy saving.

#### 3.3.1. A Priority-Based Cross Layer Routing Protocol (PCLRP)

**Figure 9:**
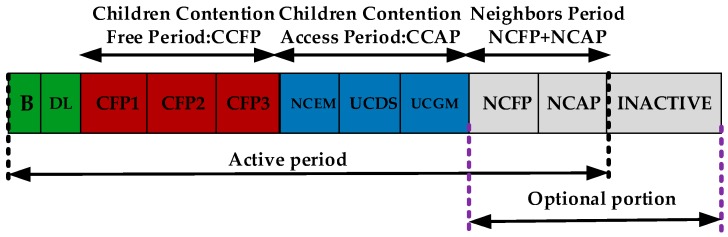
Superframe structure of the Priority-Based Cross Layer Routing Protocol (PCLRP) protocol.

A PCLRP was proposed by Elhadj et al. [25], to design the protocol across the MAC (Medium Access Channel) layer and the network layer. This paper divides data into three categories and sets priority—P1 is emergency data (EM), referring to abnormal data; P2 is delay sensitive data (DS), referring to medical video; P3 is general data (GM), corresponding to the normal cycle data. The MAC superframe division stage is shown in Figure 9. The children competition access phase (CCAP) and children competition free phase (CCFP) are divided into three corresponding slots, according to priority, and the service is customized for different priority data. In order to ensure the priority transmission of P1 data, EM data are required to compete for access in the whole CCAP. Random back-off strategy, based on priority, is adopted in the whole competition stage, and the back-off time is determined, according to priority, so as to ensure the non-delay transmission of emergency data.

In the routing phase of the PCLRP protocol, the co-operative transmission mode of relay is adopted. In the beacon phase, the coordinator judges whether the node is a relay node by the ACK information, which includes the location, energy, and current available time slot of the node. In the routing establishment phase, path planning is carried out according to data priority and the MAC slot, which achieves higher performance than a single layer protocol, and especially guarantees low delay and high reliability transmission of high priority data, and reduces conflicts and delays caused by competition. However, the protocol cannot effectively guarantee the reliable transmission of low priority data.

#### 3.3.2. Cross-Layer Design for Optimizing Transmission Reliability, Energy Efficiency, and Lifetime (CLDO)

Chen et al. [26] proposed a cross-layer design optimization scheme CLDO, which optimizes network parameters, such as transmission reliability R_i_, energy efficiency χ and network lifetime T_life_, through cooperation among network layer, MAC layer, and the PHY layer. The goal is to find the best transmission power, the best relay and the best packet size to solve the above problems. Before the theoretical deduction, the author carried out simulation experiments, the results showed that an appropriate transmission power and packet size can not only ensure a reliable transmission, but can also save energy consumption and prolong network life. The best relay can ensure a successful delivery of data packets and reduced errors, and energy consumption can be greatly reduced within an acceptable delay range. Therefore, based on this experiment, a large number of complex theoretical deductions have been carried out, and more convincing corresponding propositions and proofs have been given. In the routing stage, when selecting the next hop relay, the power efficiency and energy consumption rate of the node were considered comprehensively. When the residual energy of the node was lower than a certain value, the node only transmitted its own information, and readjusted the transmission power and packet size, to protect the node with low residual energy, ensure reliable transmission, and prolong the life of the whole network.

#### 3.3.3. Cross-Layer Retransmit Strategy (CLRS)

**Figure 10:**
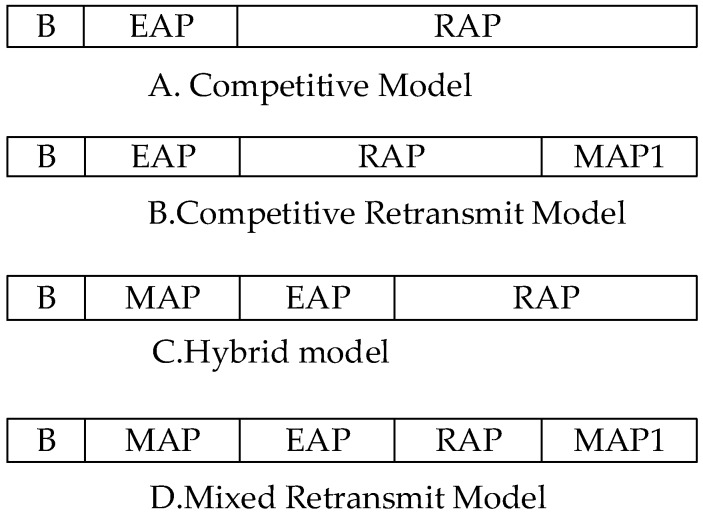
CLRS Protocol Superframe Classification.

Wang and Guo [27] proposed a cross-layer retransmit protocol CLRS, based on the IEEE 802.15.6 standard, which was different from other cross-layer methods, this protocol was designed to retransmit data packets that failed to transmit. In this study, collision and shadow effects were the two main reasons for data transmission failure, and different waveforms the appeared on the output waveforms of the receiving end. The reason of the failure was judged by the characteristics of the waveforms of the data frame, and then the method of retransmitting in MAC was decided. The superframe structure is shown in Figure 10.

The B and D modes of the superframe is specially used for low priority data in the MAP1 phase, to avoid high priority data encroaching on transmission resources. The use of TDMA to access channels in the MAP1 phase of the low priority frame, can increase the transmission success rate and reduce the transmission failure rate. For data failure caused by collision, the current time is judged to be in the period of the superframe, and the retransmission resources are allocated according to the superframe model. If the transmission fails in the MAP phase, the data is retransmitted in the subsequent competitive period; if the transmission fails in the EAP or RAP phase, then the data is re-competed and retransmitted, and the random back-off time is determined by the priority of the data. For the communication failure caused by human shadow, the sink node informs the data sending node to suspend data retransmit and wait for the shadow effect to end, before the data retransmission.

#### 3.3.4. Cross-Layer Optimization Based on Prediction

Wang et al. [28] proposed a cross-layer optimization scheme based on link quality prediction. The author believes that all human activities have periodicity to predict the network topology, in order to design a more efficient routing, which is indeed worth affirming. Considering the capability difference between the nodes and the coordinators, two kinds of link quality prediction methods, with different complexity, have been proposed, for the links between two nodes and the links between nodes and coordinators, respectively, as shown in (7) and (8):

(7) $${\hat{R}}_{ij}(t)=\beta {\hat{R}}_{ij}(t-1)+(1-\beta )\sum_{k=t-W_{T}}^{t-2}R_{ij}(k)$$

(8) $${\hat{R}}_{is}(t)=R_{is}(t)-{\mathrm{ECV}}_{t}$$

where $R_{is}(t)$ is the instantaneous link quality between node i and coordinator s, ${\mathrm{ECV}}_{t}$ is the error correction value at time t, and β is the weight factor, which coordinates the weight of historical data and the latest predicted value.

In order to better adapt to the mobile network environment, the protocol exploits the relay multi-hop transmission mode. Considering that the fixed transmission power in the mobile environment inevitably results in energy waste or data packet loss, this study adopted an adaptive adjustment of the transmission power of nodes. By comparing the predicted value of link quality with the standard value, when the predicted value was larger than the standard value, the transmission power was reduced to save energy, and vice versa. Moreover, considering the data priority, combining the random back-off time of MAC layer and increasing the transmission power to ensure reliable transmission of high priority data.

#### 3.3.5. Joint Transmission Power Control and Relay Cooperation (ATT)

Zhang et al. [29] proposed a cross-layer scheme of adaptive transmission power, based on a autocorrelation coefficient ATT, a joint transmission power control, dynamic slot scheduling, and a two-hop cooperative transmission mechanism, which realized the trade-off between reliable transmission and energy. The scheme consisted of three stages—channel state prediction, adjustment of transmission power, rearrangement of slot sequence for direct transmission, and selection of relay nodes. At the beginning of the protocol, the channel states of all nodes were calculated by hub nodes. The channel state gains were subject to the Gauss random distribution. The formulae is as follows:

(9) $$G_{i}(S+1){|}_{G_{i}(S)}\sim N\left((1-\rho_{i})\mu_{i}+\rho_{i}G_{i}(S),(1-\rho_{i}^{2})\sigma_{i}^{2}\right)$$

In which the state of the next superframe channel S+1 could be estimated by $\left(1-\rho_{i})\mu_{i}+\rho_{i}G_{i}(S\right)$.

**Figure 11:**
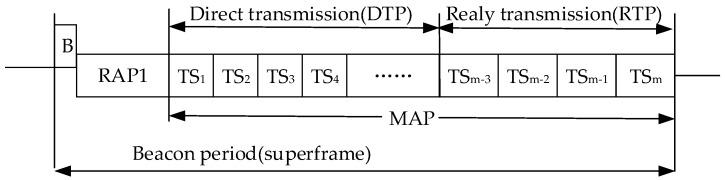
Superframe structure of the ATT protocol.

Hub node adaptively adjusts the transmission power, according to the estimated channel state, to achieve the purpose of energy saving. Then the slot order of DTP in direct transmission phase was rearranged, based on the channel state. The MAC superframe structure of ATT protocol is shown in Figure 11. The RAP1 phase is accessed by the CSMA/CA (Carrier Sense Multiple Access with Collision Avoidance) mechanism, and the MAP phase is divided into direct transmission phase DTP and relay transmission phase RTP. Each node completes information transmission in the corresponding slot. The node with relay capability maintains the receiving state at the DTP stage, and if there is information to be forwarded, then it completes the forwarding in the corresponding slot of the RTP stage. The slots with good channel state are arranged in front, after the channel state estimation is completed. In order to avoid a node acting as a relay node, many times, the protocol regulations that the predicted channel gains, are all candidate relay nodes when they are higher than the standard value. Finally, a relay node is selected randomly from the candidate. Combined with the above methods, the protocol achieves a relatively good reliability transmission, compared with other protocols. At the same time, the energy consumption is greatly reduced through adaptive power regulation. The dynamic slot scheduling method also improves the data delivery rate, relatively.

#### 3.3.6. Comparison and Analysis

As shown in Table 4, these protocols are optimized across two or three protocol layers. In terms of reliability metrics, CLDO [26], Wang et al. [28], and AAT [29] protocols have advantages. CLDO achieves reliability transmission through complex theoretical analysis, but complex computation in the initial stage of the protocol, inevitably brings additional delay and fails to achieve a performance balance. PCLRP [25], CLRS [27], and Wang et al. [28] all consider the priority of data, and both PCLRP [25] and Wang et al. [28] use back-off time mechanism in the MAC layer, which guarantees a low delay and a reliable transmission of high priority data. The difference is that Wang et al. [28] designs an adaptive power control in the physical layer, taking into account the reliability of low priority data transmission, reducing the delay of data transmission, so it is slightly better than PCLRP, in reliability and delay. The disadvantage of CLRS [27] is that when the shadow effect occurs, the whole transmission stops, until the shadow disappears, which inevitably causes a great delay.

Adaptive power control is a hot topic in current research. Both Wang et al. [28] and AAT mention adaptive power control. AAT is based on the estimation of channel state, while Wang et al. [28] is based on the prediction of link quality. Each method is worth learning, its common point is to achieve the purpose of reliable transmission and energy efficiency by power control. However, the disadvantage of Wang et al. [28] is that the selected relay node is always used until the energy of the node is lower than a certain value or the channel quality deteriorates, which inevitably leads to the early death of the node and affect the lifetime of the whole network. Based on the above analysis, AAT protocol is more balanced in reliability, delay and efficiency, and is more perfect if data priority is taken into account.

Cross-layer method plays an important role in the overall optimization of network. WBAN needs not only the superiority of a single performance, but also the imperfection of all performance, it can achieve ideal results by trade-offs among multiple performances.

### 3.4. Cluster-Based Routing

Cluster-based routing is a method borrowed from WSN. The LEACH (Low Energy Adaptive Clustering Hierarchy) routing protocol is a classic work in WSN, and the cluster-based routing method comes from it; many experiments have proved that the clustering method is more suitable for WBANs. When the number of nodes increases and the relative distance between the nodes increases, the clustering method can ensure network connectivity, balance the energy consumption of the network center and edge, adapt to the dynamic topology structure, and improve the robustness of the network. Clustering routing protocol divides the nodes in the network into clusters of nodes. Each cluster consists of several cluster nodes and a cluster head. The cluster head is elected by algorithm and is responsible for integrating and forwarding the information in the cluster, to reduce the overhead of direct communication.

#### 3.4.1. Dual Sink Approach Using Clustering (DSCB)

Ullah et al. [30] proposed a cluster-based dual sink node routing protocol DSCB, its greatest innovation is the use of dual sink nodes. Through routing analysis of some single sink. This study found that there were many shortcomings, such as congestion, high data transmission failure rate, limited coverage, and NLOS communication, which could not adapt to a dynamic environment. In the actual application of WBAN, limb movement causes a shadow effect, and the transmission interruption caused by shadow effect needs to wait for it to disappear, before it can continue to transmit, which causes a great transmission delay. Once the interruption occurs in the emergency data period, it causes transmission failure and endangers life. The method of double sink can improve the negative consequences of the shadow effect, balance the network load of a single sink node, and improve the practicability of the WBAN.

DSCB protocol fixes the nodes in the network, and the two Sink S_1_ and S_2_ are located on the front and back of the human body, respectively, which are defined as cluster head (CH), in advance. Cluster 1 and Cluster 2 are composed of other nodes on the front and back of the human body, respectively. When the node has emergency data, it communicates directly with the corresponding CH node. If it is general data, it transmits through a multi-hop mode. The cost function CF is used to select the next hop node. Considering the distance, residual energy, and transmission power, the formulae is:

(10) $$C.F_{n}=\frac{d}{E_{Residual_{n}}*T.P_{n}}$$

where d represents the distance between the node and the sink, $E_{Residual_{n}}$ represents the residual energy of the node, and T.P represents the transmission power. The simulation results show that the protocol reduces the transmission delay, improves the stability of the network, and improves the energy efficiency and reliable transmission.

#### 3.4.2. An Energy Efficiency Routing Protocol (CRPBA)

Bahea et al. [31] proposed a cluster-based routing protocol CRPBA, which referred to sink nodes as a gateway node. The nodes in the network choose direct communication or cluster-based communication, according to the distance between the gateway and node or data type. The two gateway are located at the waist and neck, respectively. The protocol stipulates that some key data communicate directly with the appropriate gateway node, to reduce transmission delay. The common data are transmitted to the cluster head by the cluster members, and then forwarded to the gateway by the cluster head, so as to reduce energy consumption and improve the success rate of data transmission. With regard to the choice of cluster head, the protocol uses cost function to decide that the node with the lowest cost function is the cluster head, and the cost function is as follows:

(11) $$Function(d,E)=\frac{distance}{Energy}$$

where the distance refers to the distance between gateway and source node, the Energy refers to the residual energy of the node. In a sense, the protocol also adopts the method of double sink, which can theoretically achieve a better performance than a single sink, but the method of choosing cluster heads is too simple, considering only distance and residual energy, the cluster heads of the near gateway consumes too much energy and dies in advance, thus, affecting the performance of the whole network.

#### 3.4.3. Comparative Analysis

There are many similarities between the DSCB and CRPBA protocols. As shown in Table 5, both sinks are used to balance the load and energy consumption of single sink and improve network performance. In DSCB [30], cluster head is pre-designed, considering the residual energy of nodes, transmission power and distance between sink, to choose the next hop. Whereas, the CRPBA [31] uses the residual energy of nodes and distance between sink to select the cluster head. The two-hop transmission also transfers data through a cluster head. Compared with the DSCB protocol, the energy efficiency of the CRPBA cluster head is slightly deficient. In theory, cluster-based protocols can solve network hotpot issues, well, but neither of them can solve the problem, the problem lies in the methods of cluster head selection and cluster size determination. However, the research of this problem in WSN has been quite mature, and can be properly introduced into WBAN research.

Compared with other routing methods, the research on cluster-based routing methods is not very mature. Some existing studies usually refer to the methods of WSN, which are not suitable for WBAN. The research on WBAN is still insufficient and is not perfect, there is still a lot of research space for cluster-based routing in the future. The problem that needs to be solved is how to choose the appropriate cluster head and determine the size of cluster, to realize the real balance of network energy consumption, improve the success rate of data transmission, and solve the reliability transmission of the edge nodes and network hotpot issues.

### 3.5. Qos-Based Routing

Qos-based routing plays an important role in any application technology, especially in resource constrained WBAN, which is a huge challenge. The Qos that need to be considered in the WBAN are—data priority, energy efficiency, link reliability and data transmission reliability, low transmission delay, node temperature, data security, etc.

#### 3.5.1. Designing Lightweight QoS Routing Protocol (LRPD)

**Figure 12:**
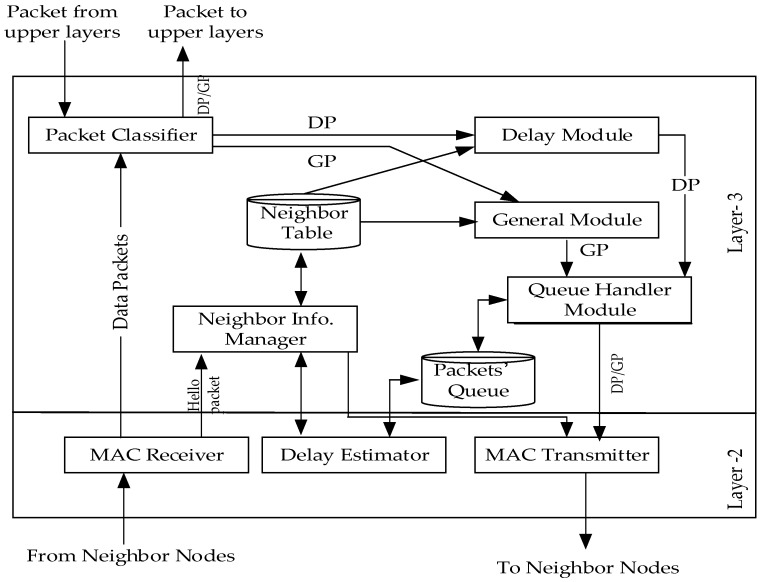
Modular diagram of the LRPD protocol.

The LRPD protocol proposed in Kuma et al. [32] is specially designed to optimize delay Qos, the protocol adopts a modular approach, and the modules closely cooperate to achieve goal optimization, as shown in Figure 12. The data from the upper layer are first entered into the data classification module, for priority division. The data are divided into general packets (GP) and delay sensitive packets (DP). The highest priority belongs to DP. Then, the DP and GP data are entered into the general module and the delay module, respectively, and are eventually sent to the queue processing module. The high priority data need not wait for direct transmission, while the low priority data need wait until an appropriate transmission time, in order to ensure the non-delay transmission of DP data.

The LRPD protocol theoretically analyses the total delay, the possible total delay from the source node to the destination node is as follows:

(12) $$D^{tot}=D^{q}+D^{tr}+D^{pr}+D^{proc}$$

(13) $$minimize(D_{ij}^{tot}),\forall j\in NH_{i}$$

where $D^{q}$ is the queue delay, $D^{tr}$ is the transmission delay, $D^{pr}$ is the transmission delay, $D^{proc}$ is the processing delay, $NH_{i}$ is the next hop set of node *i*. The simulation data prove that the protocol reduces the end-to-end delay and meets the Qos requirements.

#### 3.5.2. Hybrid Data-Centric Routing Protocol (HDPR)

**Figure 13:**
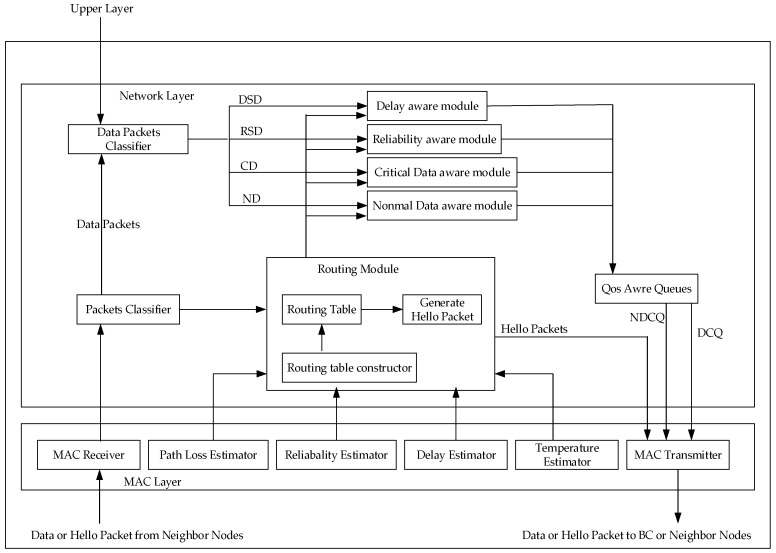
Modular diagram of the HDPR protocol.

Vetale et al. [33] proposed a data-aware hybrid routing protocol HDPR, which adopts a modular approach. As shown in Figure 13, the design of Qos varies across the network layer and the MAC layer. Data are entered by the receiver into the MAC layer, and then sent to the data classification module, which divides the data into delay sensitive data (DSD), normal data (ND), critical data (CD), and reliability sensitive data (RSD). Data priority is CD, DSD, RSD, and ND, from high to low. Data are classified into the Qos perception module, through their respective data modules. The task of this module is to send them to the corresponding module, according to the needs of each module. For example, DSD data is sent to the delay estimation module, and then the routing module finds the best path for them. This protocol mainly considers the Qos indexes, such as path loss, link reliability, delay, and temperature. Its greatest advantage is that it takes the above Qos into account and carries out the overall optimization design. Moreover, the relay node used in this protocol, only has the function of receiving and sending, which reduces the energy consumption of the acquisition node and prolongs the network life.

#### 3.5.3. Comparison and Analysis

As shown in Table 6, both LRPD [32] and HDPR [33] protocols adopt modular methods to optimize Qos. LRPD is designed to optimize the network metrics for delay, considering all delays in the whole process, and then minimize them to reduce the overall network delay. HDPR considers Qos metrics, such as delay, reliability and node temperature, synthetically, and the modular method has a clear division of labor and close cooperation, it not only achieves a low delay and high reliability, but also improves the problem of node temperature rise. In terms of energy efficiency, HDPR adds additional relay nodes and stipulates that relay nodes only have receiving and forwarding functions, which can balance the energy consumption of biological nodes. While LRPD uses minimum hop routing, which is bound to result in a high energy consumption, due to the large distance. Therefore, the above analysis shows that HDPR is more perfect and stable than LRPD.

### 3.6. Routing of Other Methods in WBAN

#### 3.6.1. Multipath-Based Routing

**Figure 14:**
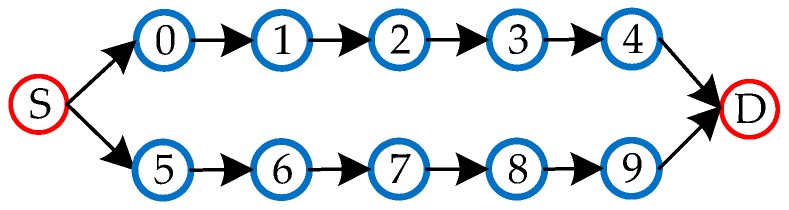
Node independent multipath routing.

In addition to the above routing methods, some researchers have introduced the WSN multipath routing method into the routing design of WBAN, by generally, using the node-independent multipath routing method [34,35]. Multipath refers to the simultaneous establishment of multiple transmission paths, between the source node and the destination node, to transmit data in groups, in parallel. While node-independent multipath routing refers to the independent paths between the source node and the destination node, and there is no common node or path between them, as shown in Figure 14. The advantage of this method is that the multipath can be used to ensure a stable transmission, balance energy consumption of nodes, and prolong network lifetime, but there might be shortcomings when data failure in a packet might affect the accuracy of the whole data.

#### 3.6.2. Mobile Sink-Based Routing

Mobile sink based routing is also a method referred from WSN, but there are only a few studies on WBAN, based on this method, at present. Mobile sink refers to sink nodes in the network that can move randomly, and the direction, speed, and residence time of its movement are pre-set, while the general WBAN stipulates that the location of the sink is fixed. Generally, the nodes in the network center consume too much energy due to the forwarding of information to their neighbors, which is called a hotspot. On the other hand, the nodes on the edge of the network consume too much energy, because of distance, which also leads to premature death and affects the connectivity of the network. The existing methods cannot solve the above problems, very well, but the mobile sink-based method can relatively alleviate the “energy hole” caused by hotspot and the “coverage hole” caused by edge nodes, and reduce the delay and energy caused by multi-hop. It can greatly promote the further development of WBAN.

## 4. Prospects for WBAN and Suggestions for Routing Design

In the future, the development of WBAN will be explosive. With the rapid development of Internet of Things, artificial intelligence, and the coming 5G era, WBAN will play a decisive role in human society. Everyone is looking forward to the application of WBAN as soon as possible, but there are still many problems to be considered at present [36,37]. The limited energy of the nodes is an important factor hindering the development of WBAN, thus, energy efficiency must be considered. We cannot only rely on energy harvesting technology, but should also explore the energy efficiency method from the source. In the future, the energy efficiency routing design of WBAN can, not only be improved and optimized, but should also be bold to innovate new methods and new ideas. The design of energy efficiency routing can reference the following ideas. 

### 4.1. Prospects for Current Routing Design

- In the analysis of the above routing methods, both MHRP [18] and HDPR [33] protocols, stipulate that the relay node only has the function of receiving and forwarding, and the biological node only collects and transmits information, without forwarding other information. This method can reduce the energy consumption of the biological node and prolong the network life [38]. However, the problems are—how to determine the number and location of additional relay nodes? Will the connectivity of the network topology be guaranteed after its death? Whether the added nodes will affect people’s comfort and so on. WBAN is a technology applied to human body, the above issues need to be carefully considered.
- In the routing design, the hottest research at present is about the next hop selection method [39,40,41,42], which has both optimization and innovations. For example, the literature [22,23,30] uses the cost function to solve the next hop selection. In recent years, the cost function has been developed from a single parameter to multi-parameters, and the weighting factor has been used to weigh the multi-parameters, which achieves a better performance than a single parameter. It is believed that the cost function plays a more important role in the design of energy efficient routing in the future.
- The cross-layer method can achieve a better performance than a single-layer, and can achieve unexpected results in energy efficiency. Cross-layer is a comprehensive design of performance by coupling multiple protocol stacks, such as combining network layer, MAC layer, and physical layer, planning appropriate transmission power in physical layer, efficient slot allocation technology in MAC layer, and reliable routing design in network layer, which can theoretically achieve better network performance, reduce energy consumption of nodes and realize energy efficiency of the whole network.
- Postures-based methods can make full use of mobility, in terms of energy efficiency. If the various postures of human body can be skillfully utilized, then the delay and energy consumption can be greatly reduced in theory. Posture prediction can be used to select routing quickly and reduce energy waste in the routing process, but the premise is to solve the problem of human posture recognition, which requires joint efforts in many fields.

### 4.2. Prospects for Future Routing Design

In the future routing design, we can put aside the existing methods to explore and innovate in new areas. In essence, WBAN is a special application of WSN and also a branch of WSN, so it is natural to learn from the relevant energy efficiency technology of WSN, and then optimize and innovate, according to the particularity of WBAN. Since the development of WSN, there are many energy efficiency routing designs and the technologies are mature and perfect. Learning from the relevant technologies in WSN can greatly promote the rapid development of WBAN. For the energy efficiency routing design, we can draw on following methods and ideas.

- Cluster-based approach: At present, there are many technologies about energy efficiency design in WSN, and the cluster-based routing is the hottest research. This method is no longer restricted to the improvement of traditional methods, but integrates various intelligent theories and algorithms. For example, Jiang et al. [43] proposed a hybrid routing algorithm, which combines ANT colony optimization algorithm and minimum hop routing, to find the optimal path, with the minimum energy consumption and the fastest speed. This method not only improves the ANT colony algorithm, but also optimizes and innovates the minimum hop routing, and it has a great improvement in energy efficiency and delay. Morsy et al. [44] proposed an efficient routing algorithm, the process of CH selection is formulated as a single-objective optimization problem to find optimal set of CHs to form, one-hop clusters, in order to balance energy consumption, enhance stability, and scalability, using gravitational search algorithm (GSA). This protocol solved the problem of energy hole, coverage hole, and balanced the network energy consumption. In addition to the above methods, there are many other similar intelligent methods [45,46,47,48,49], such as distributed artificial ANT colony algorithm, circular random firefly algorithm, particle swarm optimization algorithm, and so on. However, at present, the intelligent methods in WBAN is limited [50,51,52,53,54], and there is a lot of space for research in the future. We cannot copy the method of WSN completely, but should consider the Qos requirement of WBAN and improve it properly. In routing design, we should also consider the minimum hops, the lowest delay, the temperature of the nodes, and the movement of the limbs. Only suitable for WBAN is the real reference.
- Data aggregation method: It is also an energy efficiency design from the source. Data aggregation can effectively reduce redundant data transmission and node energy consumption, and improve the energy utilization of nodes. Although there is no large-scale research on data aggregation technology for WBAN, data aggregation method is a really good way to save energy. Therefore, we can reference the data aggregation method from WSN [55,56], which need more scholars to study and explore in the future.
- Mobile sink-based routing. In recent years, the use of mobile sink has drawn enormous attention for data collection in WSNs, so there are many relevant research studies on this area [57,58,59,60]. For example, Gharaei et al. [59] proposed a mobile sink (MS)-based inter- and intra-cluster routing algorithms, which is a routing strategy specially designed for the problem of energy hole and coverage hole. The algorithm controls the sojourn position and stay time of mobile sink, effectively solving the above two problems. Zhang et al. [60] proposed a dynamic path planning, based on mobile sink for emergency traffic, this protocol considers the priority of data, as well as the emergency traffic perception and estimation, to dynamically adjust the routing and ensure the reliability of data transmission. Therefore, the application of mobile sink in WSN indeed solves many bottleneck problems, such as “energy hole” and “coverage hole”. Similar bottleneck problems also exist in WBAN, so the introduction of mobile sink into WBAN can theoretically alleviate or solve the above problems. At present, the research about this method in WBAN is just at the initiation stage. Thus, future research on this method must refer to WSN. The problem to be considered is the selection of an optimal path, and specific application scenarios need to be treated in detail. The specific application of mobile sink in WBAN still needs the efforts and exploration of other scholars in the future.

In addition, when processing heterogeneous data of WBAN, relevant technologies of DTN can also be combined. The most famous technology is “storage-carry-forward”, which is suitable for an environment of unstable connection between nodes and intermittent topology, which is very similar to the dynamic scene of WBAN. At present, there is still no good solution to deal with the dynamic scene, because the movement of the human body leads to topology separation or shadow effect. At this time, the “storage-carry-forward” method of DTN can be used to solve the problem of data loss during topological separation, while this method is not suitable for an emergency data processing. Therefore, we should consider its applicability and the particularity of WBAN, when referring to other routing methods.

The future progress of WBAN is bound to change with each passing day, and its application scenario must be complex and changeable. This paper only reviews the routing methods of single WBAN, which is based on narrowband communication, under the standard of IEEE 802.15.6. But nowadays, more and more research for WBAN, based on IR-UWB [61,62,63,64,65], IR-UWB communication can achieve a short-distance high-speed data communication, at very low power consumption, which has a great potential in energy efficiency routing design of WBAN. In addition, this paper did not study the topological environment of multi-WBAN, which may have some limitations, but the research of single WBAN is the first step to realize the application. Only by solving the technical problems of single WBAN, can the real interconnection be realized. Therefore, it is of great significance to review the routing methods of single WBAN.

## 5. Conclusions

In this survey, various existing routing protocols that are proposed in WBANs were categorized and detailed analyzed. It was seen that the routing protocol plays an important role in the design of energy efficient, reliable and low cost WBANs. Based on the method and the design target, the routing protocols for WBANs were categorized as postural-based, temperature-based, cross-layer, cluster-based, and Qos-based routing. Moreover, a comparison of different protocols has been analyzed so that an appropriate protocol can be selected, according to the targeted application. This survey will benefit the researchers to study the energy efficient routing protocols for WBANs in the field of healthcare systems.

## Figures and Tables

**Table 1:** Comparison between WSN and WBAN.

Problem	WSN	WBAN
range	environmental monitoring(m/km)	body range(cm/m)
number of nodes	hundreds	dozens
node size	no special requirements	the smaller the better
node task	single or scheduled tasks	many
data rate	homogeneous	heterogeneous
data loss	tolerable	intolerable
node placement	easily	difficult
biocompatibility	not considering	consider
node life	months/years	the longer the better
topological	unchanged	changed
node energy	limited, but replaceable	limited and irreplaceable
safety	low	very high
standard	IEEE 802.11.4	IEEE 802.15.6

**Table 2:** Posture-based routing protocols comparison.

Protocol	Goal	Characteristic	Complexity	Delay	Energy Efficiency
MHRP (2017)	Dynamic environment Cardiac monitoring	A fault-tolerant system consists of two identical and independent sets of nodes	low	low	high
NCMD (2017)	Dynamic environment Topological fracture treatment	Opportunities to establish connections, Minimizing network management	high	low	high

**Table 3:** Comparison of temperature-based routing protocols.

Protocol	Goal	Characteristic	Complexity	Delay	Energy Efficiency
TARA (2005)	temperature	withdraw strategy to avoid high temperature nodes	low	high	low
ER-ATTEMPT (2014)	temperature	Considering temperature and hops	low	low	medium
TTRP (2017)	temperature	Considering temperature and trust	high	low	high
MTR (2017)	temperature	Considering temperature and mobility	high	medium	medium

**Table 4:** Comparison of Cross-Layer Routing Protocols.

Protocol	Cross Layer	Characteristic	Priority	Reliability	Delay	Energy Efficiency
PCLRP (2016)	MAC and network	Slot partitioning and routing customization	√	medium	high	medium
CLDO (2017)	PHY, MAC and network	Finding the best power, relay and packet size	N/A	high	medium	high
CLRS (2018)	PHY and MAC	Improvement of retransmitting superframe	√	N/A	high	N/A
[28] (2018)	PHY, MAC and network	Link quality prediction and adaptive power control	√	high	medium	low
AAT (2018)	PHY, MAC and network	Channel state estimation and adaptive power control	N/A	high	medium	high

**Table 5:** Cluster-based routing protocols comparison.

Protocol	Sink Quantity	Characteristic	Communication Mode	Delay	Energy Efficiency
DSCB (2017)	2	Next hop: $C.F_{n}=\frac{d}{E_{Residual_{n}}*T.P_{n}}$	Emergency data: single hop	low	high
			General data: multi-hop		
CRPBA (2018)	2	CH selection: $Function(d,E)=\frac{distance}{Energy}$	Emergency data: single hop	low	medium
			General data: two hops		

**Table 6:** Comparison of the Qos-based routing protocols.

Protocol	Goal	Characteristic	Priority	Delay	Reliability	Energy Efficiency
LRPD (2017)	optimization delay	Modularization	√	low	N/A	medium
HDPR (2017)	Optimizing Delay, Reliability and Node Temperature	Modularization	√	low	high	high
